# Diagnostic accuracy of the cancer ratio for the prediction of malignant pleural effusion: evidence from a validation study and meta-analysis

**DOI:** 10.1080/07853890.2021.1906943

**Published:** 2021-04-05

**Authors:** Ying Zhang, Xiaoou Li, Junhui Liu, Xueru Hu, Chun Wan, Rui Zhang, Yongchun Shen

**Affiliations:** a Department of Respiratory and Critical Care Medicine, West China Hospital, Sichuan University, Chengdu, China; b Division of Pulmonary Diseases, State Key Laboratory of Biotherapy of China, Chengdu, China; c Department of Medical Informatics, West China Hospital, Sichuan University, Chengdu, China

**Keywords:** Malignant pleural effusion, cancer ratio, carcinoembryonic antigen, diagnosis, meta-analysis

## Abstract

**Objective:**

This study aimed to assess the diagnostic accuracy of serum LDH to pleural ADA ratio (cancer ratio, CR)for malignant pleural effusion (MPE) through an original study and meta-analysis.

**Methods:**

We retrospectively collected data from 145 patients with MPE and 117 cases of benign pleural effusions (BPE). The diagnostic performance of CR and a typical biomarker of MPE, carcinoembryonic antigen (CEA), were analysed using the receiver operating characteristic (ROC) curves and the area under the curve (AUC) as a measure of accuracy. The overall diagnostic accuracy of CR was summarised by a standard diagnostic meta-analysis.

**Results:**

Significantly higher CR and pleural CEA values were observed in the MPE patients than in the BPE patients. At a cut-off value of 14.97, CR showed high sensitivity (0.91), low specificity (0.67), and high AUC (0.85). The combination of CEA and CR increased the AUC to 0.98. The meta-analysis included seven studies involving 2,078 patients. The pooled values for sensitivity, specificity, positive/negative likelihood ratio, and diagnostic odds ratio of CR were 0.96, 0.88, 7.70, 0.05, and 169, respectively. The AUC of the summary ROC of CR was 0.98.

**Conclusion:**

CR has a high diagnostic accuracy for predicting MPE, especially when used in combination with pleural CEA.

## 1. Introduction

Malignant pleural effusion (MPE) is a common clinical condition observed in patients suffering from malignant diseases, such as primary thoracic cancer, pleural mesothelioma, metastatic cancer, etc [1–3]. It is associated with unfavourable prognosis and a median survival time of 3–12 months [4–5]. The estimated annual incidence of MPE is between 150,000 and 175,000 in the US [2], and around 40,000 in the UK [3]. MPE is considered to be the first aggressive sign of malignant diseases in approximately 10% of patients [1]. For the purpose of disease staging and the development of effective treatment plans, it is important to diagnose MPE early and accurately by using minimally invasive methods; unfortunately, this continues to be a clinical challenge [6]. A number of tumour markers have been used for the diagnosis of MPE, including vascular endothelial growth factor, carcinoembryonic antigen (CEA), carbohydrate antigen (CA) 125, CA 15-3, CA 19-9, and CYFRA 21-1 [7–9]. However, none of these markers has shown both high sensitivity and high specificity. Therefore, it is imperative to identify a novel marker to improve the accuracy of MPE diagnoses.

Lactate dehydrogenase (LDH) is a ubiquitous enzyme that occurs in high concentrations in the liver, kidney, myocardium, skeletal muscles, and red blood cells [10]. It plays an important role in glycolysis and gluconeogenesis; an increase in LDH levels can occur as a result of pernicious anaemia, shock, and tumour metastasis [11]. Previous studies have reported that elevated plasma LDH can be used as a diagnostic and prognostic marker to detect sepsis and cancer [12–15]. However, its diagnostic potential as a biomarker for MPE has not been evaluated in detail.

Adenosine deaminase (ADA) is a known biomarker of benign tuberculous pleural effusion [16,17]. It is secreted by mononuclear cells, lymphocytes, neutrophils, and red blood cells that play an important role in purine nucleoside metabolism [16,17]. Patients with MPE typically show low levels of ADA [16], but whether this can aid MPE diagnosis is unclear. One study reported that patients with MPE had a high serum LDH to pleural ADA ratio (cancer ratio, CR), yield sensitivity and specificity of 0.98 and 0.94 in diagnosing MPE, suggesting that this ratio can be used as a novel marker for MPE [18]. Although several studies have been performed to validate these findings, the results have been inconsistent, so we do not have a clear understanding of the diagnostic performance of the CR for MPE. Here we conducted a retrospective study and meta-analysis of relevant literature to evaluate the diagnostic accuracy of CR as a marker for MPE. We also assessed the potential diagnostic value of CEA, which is a well-known biomarker of MPE.

## 2. Methods

### 2.1. Patients

In this retrospective study, we considered 261consecutive patients suffering from pleural effusion who were admitted to our hospital for further investigation between February 2015 and October 2015. We included all patients who had received a confirmed diagnosis of benign pleural effusion (BPE) or MPE and who had completed routine laboratory tests associated with pleural effusion, as well as tests of serum LDH, pleural ADA, and pleural CEA. Patients with inconclusive final diagnosis and incomplete data were excluded from this study. On admission, all patients signed the informed consent for their anonymized clinical data to be analysed and published for scientific research purposes. This study was approved by the Ethics Committee of our hospital and was conducted based on the principles outlined in the Declaration of Helsinki.

### 2.2. Diagnostic criteria

In this study, MPE was diagnosed based on the presence of malignant cells in pleural effusion or pleural biopsy specimens [2–3]. In patients with BPE, tuberculous pleural effusion was diagnosed if acid-fast bacteria could be cultured from pleural fluid or sputum, or if granulomas were present in pleural biopsy specimens, or if patients responded well to anti-tuberculosis therapy during follow-up of at least 3 months. Parapneumonic effusion was defined as any effusion associated with bacterial pneumonia, lung abscesses, or bronchiectasis. Two clinicians (YZ and XL) independently evaluated the association between pleural effusion and other comorbidities, such as acute pancreatitis, based on medical history, physical examinations, computed tomography, and patients’ response to treatment.

### 2.3. Data collection and statistical analysis

We collected data on age, sex, and routine biochemical analysis results on admission, includingcolourof pleural fluid; levels of pleural glucose, proteins, ADA, and CEA; and serum LDH. Patient demographic data and disease characteristics were summarised and expressed as mean ± standard deviation (mean ± SD). Intergroup differences were assessed for significance using the Mann-Whitney U test. We analysed the receiver operating characteristic (ROC) curves and the area under the ROC curves (AUC) to assess the overall diagnostic performance of CR and pleural CEA. The AUCs of different indexes were compared using the nonparametric approach [19]. Further, the cut-off value was selected based on the best diagnostic efficacy having achieved equilibrium between sensitivity and specificity by using Youden’s index. All statistical analyses were performed in SPSS 21.0 (Chicago, IL, USA), all tests were two-sided and differences associated with *p* < .05 were considered statistically significant.

### 2.4. Meta-analysis

We systematically examined the PubMed, EMBASE, Web of Science, Chinese National Knowledge Infrastructure, Wanfang, and Weipu databases to identify studies on MPE published before June 2020. The following search terms were used in each database: “malignant pleural effusion”, “malignant pleural fluid”, “lactate dehydrogenase”, “LDH”, “adenosine deaminase”, “ADA”, “cancer ratio”, “sensitivity”, “specificity”, and “accuracy”. We included original clinical research articles that reported true positive (TP), false positive (FP), false negative (FN), and true negative (TN) data with respect to the use of CR for MPE diagnosis. Only articles published in English and Chinese were considered. Studies were excluded from this meta-analysis for the following reasons: (1) there were on data of sensitivity and specificity; (2) they were not based on human subjects; (3) Conference proceedings and studies published only as abstracts.

All records were imported into Endnote for further review. Two reviewers (YZ and XL) independently evaluated the studies initially based on titles and abstracts and subsequently based on full text. After removing inappropriate and duplicate studies, the reviewers assessed the quality of the included studies using a revised version of QUADAS-2 [20].

The indices of test accuracy, sensitivity, specificity, positive likelihood ratio (PLR), negative likelihood ratio (NLR) and diagnostic odds ratio (DOR), along with 95% confidence intervals (CIs),were pooled from each study using a bivariate model. Heterogeneity among all eligible studies was evaluated based on I^2^: an I^2^ > 50% indicated significant heterogeneity. The diagnostic performance of CR and CEA was assessed based on the summary ROC (SROC) curve. Potential publication bias was evaluated by using Deeks’ test [21]. Meta-analysis was performed in Stata 15.0 (Stata Corp, College Station, TX, USA), and *p* < .05 indicated statistical significance.

## 3. Results

### 3.1. Patient characteristics

We collected data on the clinicopathological characteristics of a total of 145 patients diagnosed with MPE and 117 patients diagnosed with BPE (Table 1). The selection process was outlined in Supplementary Figure 1. We found that a large proportion of patients with MPE suffered from lung cancer (*n* = 121), metastatic cancer (*n* = 13), hematological malignancies (*n* = 8), and pleural mesothelioma (*n* = 3). A large proportion of the BPE patients suffered from tuberculous pleural effusion (*n* = 68), followed by parapneumonic effusion (*n* = 44), hepatic pleural effusion (*n* = 2), pleural effusion due to acute pancreatitis (*n* = 2), and chylothorax (*n* = 1).

**Table 1.** Characteristics of the patients with pleural effusions in the present clinical study.

	MPE	BPE	*p* value
Number of patients	145	117	
Age (year)	61.47 ± 13.09	54.17 ± 18.67	.001
Gender			
Male	81	79	.054
Female	64	38	
Colour of pleural effusion			
Yellow	81	76	<.001
Red	53	19	
Yellow-red	11	19	
Purulent/Chylous	0	3	
Laboratory result			
Serum LDH (U/l)	215.77 ± 101.95	174.44 ± 49.03	<.001
Pleural glucose (U/l)	6.11 ± 2.39	5.50 ± 2.78	.009
Pleural protein (g/l)	41.33 ± 10.17	45.51 ± 8.13	<.001
Pleural CEA (ng/ml)	186.90 ± 271.77	1.34 ± 2.54	<.001
Pleural ADA (U/l)	8.00 ± 3.77	19.21 ± 27.59	<.001
Cancer ratio	32.96 ± 25.31	14.50 ± 9.37	<.001

BPE: Benign pleural effusion; MPE: Malignant pleural effusion; CEA: carcino-embryonic antigen; LDH: lactate dehydrogenase; Cancer ratio: serum LDH / pleural ADA.

### 3.2. Serum LDH and pleural ADA levels

Patients with MPE had significantly higher levels of serum LDH than those with BPE (215.77 ± 101.95 U/l vs 174.44 ± 49.03 U/l; *p* < .001). Conversely, pleural ADA levels were significantly higher in patients with BPE than those with MPE (19.21 ± 27.59 U/l vs 8.00 ± 3.77 U/l; *p* < .001). Therefore, CR levels were significantly higher in MPE patients (32.96 ± 25.31) than in BPE patients (14.50 ± 9.37; *p* < .001).

### 3.3. Diagnostic performance of CR and pleural CEA

**Figure 1.**
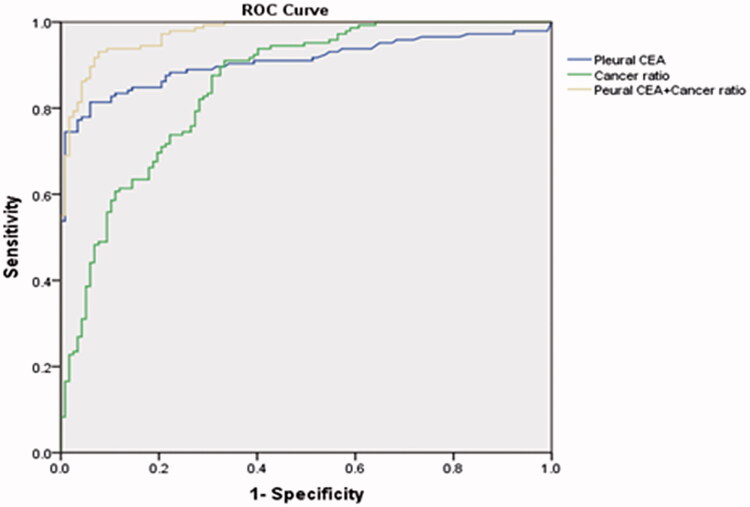
Receiver operating characteristic curves showing the performance of the cancer ratio and levels of carcinoembryonic antigen for diagnosing MPE.

ROC curves were created to summarise and compare the diagnostic performance of CR and pleural CEA as markers for MPE (Figure 1). At a cut-off value of 14.97, CR had an AUC value of 0.852. As the cut-off value raised, the specificity of the rule increased, whereas the sensitivity concurrently decreased (Supplementary table 1). With the maximisation value of the Youden index, the best cut-off was 15, along with a sensitivity value of 0.91 and a specificity value of 0.67. On the other hand, CEA had an AUC of 0.91 at a cut-off value of 2.57 ng/ml, along with a sensitivity value of 0.81 and a specificity value of 0.94. A combination of the two markers increased the sensitivity (0.93), specificity (0.92), and AUC values (0.98), which was significantly higher than CR alone (*p* < .05).

### 3.4. Meta-analysis

**Figure 2.**
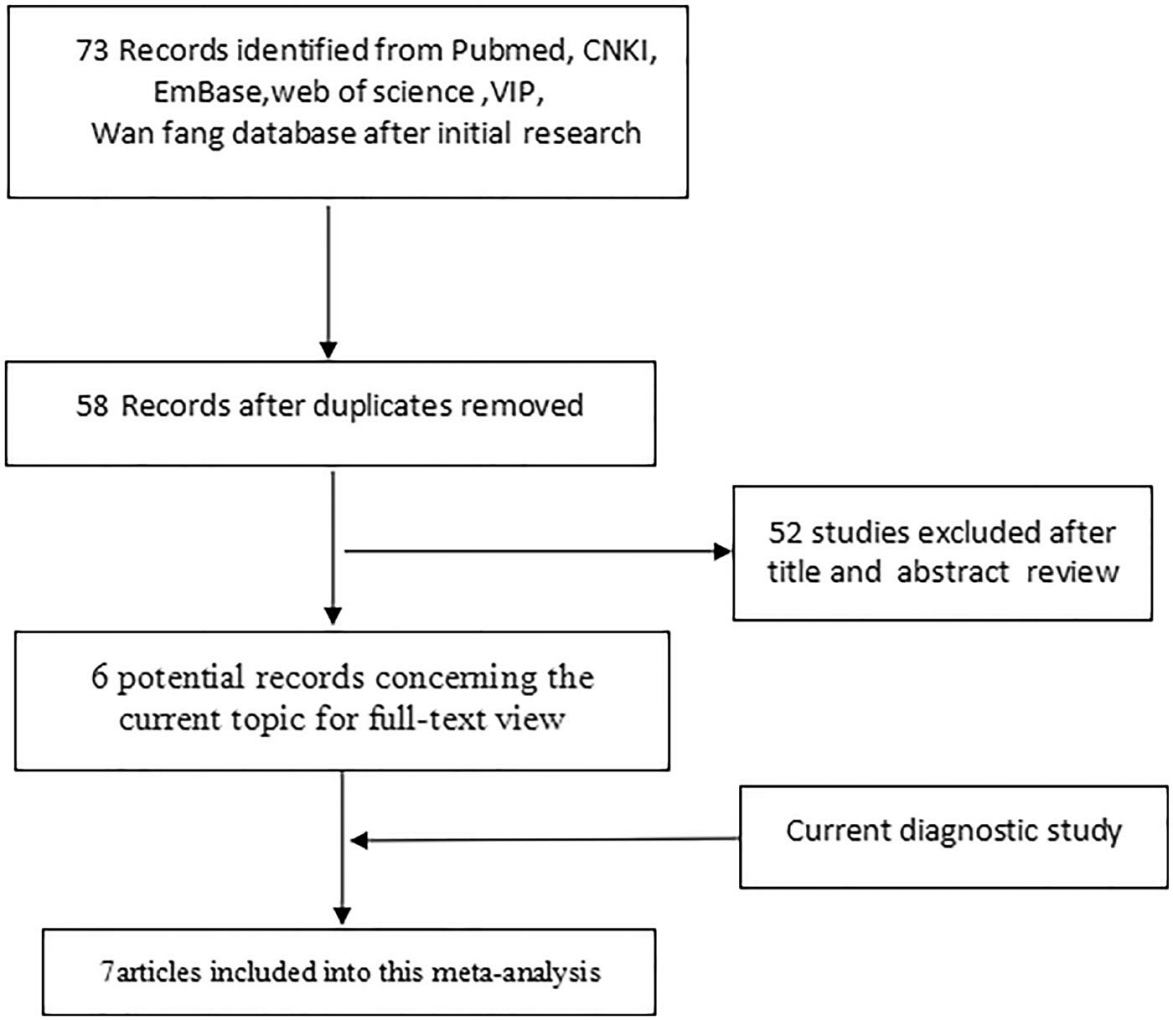
Flow diagram of study selection in the meta-analysis.

We performed a meta-analysis of 7 studies (including the present one) involving 860 patients with MPE and 1,218 patients with BPE [18,22–26]. These studies were published from 2016 to present, across five countries, three in China, two in Singapore, one in Egypt, and one in Poland. Figure 2 outlines the process of selecting studies. The clinical characteristics of the patients are listed in Table 2.

**Table 2.** Summary of eligible studies.

Author	Year	Country	MPE (*n*)	Controls	Study design	Reference	Cut-off	Sensitivity	Specificity	TP	FP	FN	TN
Verma et al. [18]	2016	Singapore	84	34	Prospective	Biopsy, Cytology	20	0.95	0.85	80	5	4	29
Verma et al. [22]	2016	Singapore	100	54	Retrospective	Biopsy, Cytology	20	0.98	0.94	98	3	2	51
Zhang et al. [23]	2016	China	318	669	Prospective	Cytology, biopsy	10.6	0.94	0.73	299	183	19	486
Elmahalawy et al. [24]	2017	Egypt	20	40	unknown	Biopsy	5.03	1	0.87	20	0	0	40
Korczyńsk et al. [25]	2018	Poland	74	66	Retrospective	Biopsy	16.4	0.95	0.68	70	21	4	45
Jiang et al. [26]	2018	China	119	238	Retrospective	Cytology, biopsy	12	0.97	0.94	115	14	4	224
Zhang et al. (Current study)	NA	China	145	117	Retrospective	Cytology, biopsy	14.97	0.91	0.67	132	39	13	78

MPE: Malignant pleural effusion; TPE: Tuberculous pleural effusion; PPE: Parapneumonic effusion; AUC: area under curve; TP: true positive; FP: false positive; TN: true negative; FN: false negative.

**Figure 3.**
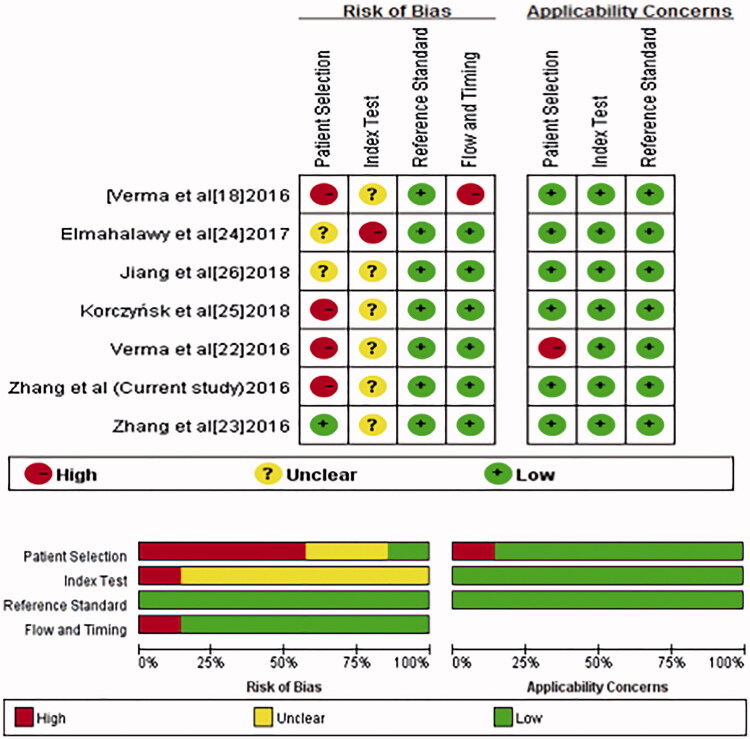
Quality assessment of studies included in the meta-analysis.

Quality assessment of eligible studies is shown in Figure 3. We observed a patient selection bias in four studies: three studies (including the present one) [18,25] used a retrospective approach and one study enrolled only patients with lymphocytic predominant exudative pleural effusion [22], For reference standard, all the studies reported the methods to diagnose MPE including cytology and biopsy. Risk of bias related to the follow-up and timing domains were high in one study [18] because patients without a final diagnosis were excluded from analysis.

**Figure 4.**
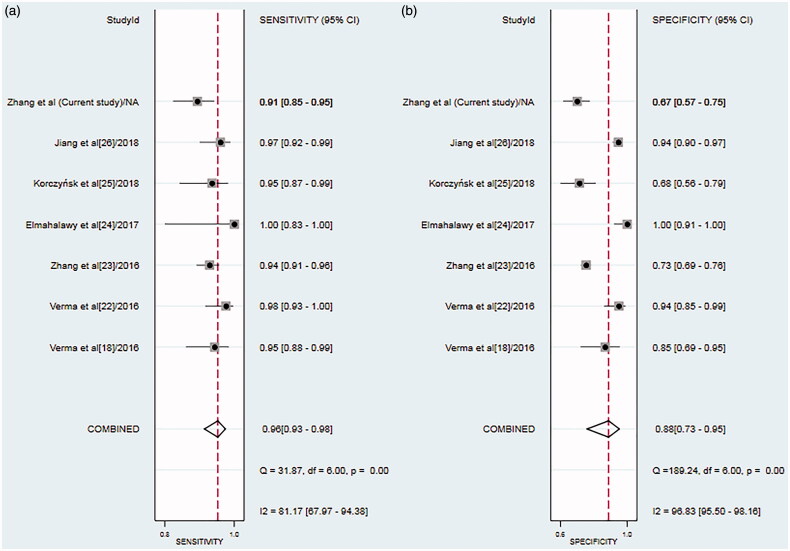
Forest plots of (a) sensitivity and (b) specificity of the cancer ratio for diagnosing MPE, along with 95% confidence intervals (CIs).

**Figure 5.**
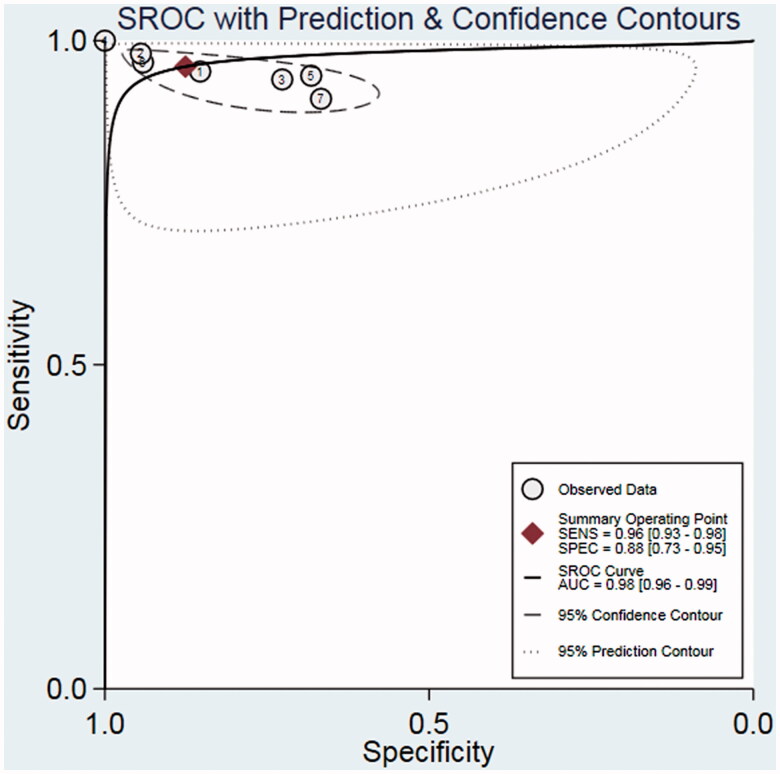
Summary receiver operating characteristic curve assessing the performance of the cancer ratio for diagnosing MPE.

To evaluate the diagnostic accuracy of CR with respect to MPE, we calculated pooled values of sensitivity (0.96, 95% CI 0.93–0.98; Figure 4(a)), specificity (0.88, 95% CI 0.73–0.95; Figure 4(b)), PLR (7.7, 95% CI 3.3–8.8), NLR (0.05, 95% CI 0.02–0.09), and DOR (169, 95% CI 39–726). The SROC curve also shows that the AUC is 0.98 (95% CI 0.96–0.99; Figure 5). These values indicate that CR is valuable for diagnosing MPE.

**Figure 6.**
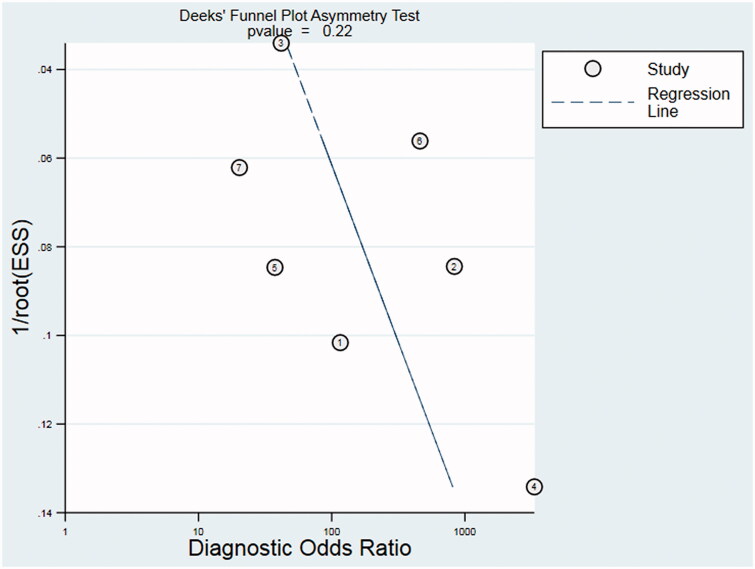
Deek’s funnel plot to assess risk of publication bias in the meta-analysis of the performance of the cancer ratio for diagnosing MPE.

Our evaluation of the included studies suggests that there was significant heterogeneity with respect to the sensitivity (I2 = 81.17%) and specificity (I2 = 96.83%) (*p* < .05). However, we could not perform a meta-regression analysis to investigate the source of heterogeneity due to the limited number of included studies. We found no significant evidence of publication bias based on the Deek’s funnel plot (*p* = .22; Figure 6).

## 4. Discussion

There is a critical need to identify a simple and effective biomarker for MPE. Studies have reported an increase in the CR in patients with MPE, suggesting that CR can be used as a novel diagnostic marker for MPE. Our study aimed to validate the diagnostic performance of CR using retrospective data collected from a Chinese cohort as well as meta-analysis of the relevant literature. Our results show that CR can be used as an effective marker for the diagnosis of MPE, especially in combination with CEA.

CR was first proposed as a predictor of MPE in 2016, when this ratio was found to be significantly higher in patients with MPE compared to those with tuberculous pleural effusion or parapneumonic effusion [18]: this study reported a cut-off level > 20 for CR, along with high sensitivity (0.98, 95% CI 0.92–0.99) and specificity (0.94, 95% CI 0.83–0.98). Our validation study of 262 patients with pleural effusion also found high sensitivity (0.91) and AUC (0.85), confirming the diagnostic accuracy of CR. However, the low specificity (0.67) indicates that although CR can be a sensitive biomarker of MPE, diagnoses based on this biomarker should be confirmed using other methods. We noticed the specificity of our study is lower than previous study [18,22], it may be caused by the different percentage of patients with MPE, aetiology of MPE, the previous studies enrolled more patients with lung cancer (95% and 97.6%) [18,22], while in our study, the percentage of lung cancer in MPE was only 83%. Since the levels of LDH may be associated with different types of tumour, thus, the clinical interpretation of CR results should consider these issues.

The results of our meta-analysis of the diagnostic performance of CR also showed high pooled values of sensitivity (0.96) and specificity (0.88). The SROC curve, which assesses overall test performance and depicts the trade-off between sensitivity and specificity, gave an AUC of 0.98, indicating a high overall accuracy. The DOR value combines sensitivity and specificity data into a single number ranging from 0 to infinity; higher DOR values indicate better discriminatory test performance [27]. The mean DOR in our meta-analysis was 169, suggesting that it is useful to consider CR levels during the diagnosis of MPE. Similarly, the pooled PLR value (7.7) suggests that MPE patients are approximately seven times more likely to have a positive CR assay result than BPE patients. The pooled NLR value (0.05) indicates a 5% likelihood that a patient whose CR is too low to qualify as MPE actually does have MPE. In fact, Han et al. also summarised the overall diagnostic performance of CR for MPE, and they only included five studies [28]. In our study, first, we performed our retrospective study to validate the previous findings; second, we enrolled more studies (total seven studies) to make a more objective conclusion. Third, we performed publication bias examination. Our study provided more evidence regarding on CR in the diagnosis of MPE.

There are several advantages with respect to the clinical utility of CR for the diagnosis of MPE. First, nearly all patients with undiagnosed pleural effusion undergo routine blood and pleural biochemical tests. Therefore, it is easy to obtain data on LDH and ADA levels, without incurring any additional costs. Second, this data can help guide treatment plans: patients with high CR values must be treated with caution, and further diagnostic examinations such as repeated cytologic test, invasive procedures such as medical thoracoscopy and pleural biopsy should be considered. While, the best cut-off value of CR has not been established, a presepcified threshold value is needed since a data-driven threshold may overstate an index test [29].

CEA has been widely used in the diagnosis of MPE. A meta-analysis based on 45 studies showed low pooled sensitivity (0.54) and high pooled specificity (0.94) for CEA when diagnosing MPE, the low sensitivity of CEA limited its role in screening MPE [7]. The present study also showed that pleural CEA is a valuable biomarker for MPE (AUC 0.91), which is higher than CR. Recent studies also identified that pleural/serum CEA ratio is also helpful for diagnosing MPE, at a cut-off value of 1, the sensitivity and specificity of CEA ratio for diagnosing MPE were 85% and 92%, respectively [30,31], and supplied additional clue for MPE. The diagnostic sensitivity of CEA is moderate, therefore, in clinical practice, the results of CEA assays should be interpreted together with clinical findings and conventional laboratory tests. In fact, the combination of CR and CEA led to higher diagnostic accuracy (AUC 0.98) than either biomarker on its own. Thus, we propose that clinicians combine CR and CEA to arrive at an accurate MPE diagnosis.

The results of our study must be interpreted with caution in the light of several limitations. Due to the retrospective nature of our study, we were unable to obtain all the relevant data required for our analysis from the hospital’s medical records, and we didn’t calculate the sample size and also can’t calculate the diagnostic performance of CEA ratio for MPE. Additionally, most of the included studies used patients with tuberculous pleural effusion and parapneumonic effusion as controls. Other aetiologies of pleural effusion, such as heart failure, chemical pleurisy, or connective tissue disease were not included. In order to extend our results, future work must include clinical data from a larger cohort of patients with many different types of pleural effusion. The limited number of studies included in our meta-analysis was also another source of bias, especially since we could not evaluate covariates as possible sources of the observed heterogeneity [32].

## 5. Conclusion

In summary, our study and meta-analysis demonstrate that CR is an effective diagnostic marker for MPE, especially in combination with CEA. Further research is required to validate these findings.

## Data Availability

All data used to support the findings of the current study are available from the corresponding authors upon reasonable request.
